# Rationale, design and methods for a staggered-entry, waitlist controlled clinical trial of the impact of a community-based, family-centred, multidisciplinary program focussed on activity, food and attitude habits (Curtin University’s Activity, Food and Attitudes Program—CAFAP) among overweight adolescents

**DOI:** 10.1186/1471-2458-12-471

**Published:** 2012-06-21

**Authors:** Leon M Straker, Kyla L Smith, Ashley A Fenner, Deborah A Kerr, Alexandra McManus, Melissa C Davis, Angela M Fielding, Tim S Olds, Martin S Hagger, Anne J Smith, Rebecca A Abbott

**Affiliations:** 1 School of Physiotherapy, Curtin University, Perth, Australia; 2 School of Psychology and Speech Pathology, Curtin University, Perth, Australia; 3 School of Public Health, Curtin University, Perth, Australia; 4 Curtin Health Innovation Research Institute, Curtin University, Perth, Australia; 5 School of Occupational Therapy and Social Work, Curtin University, Perth, Australia; 6 Health and Use of Time (HUT) Group, University of South Australia, Adelaide, Australia; 7 School of Human Movement Studies, The University of Queensland, Brisbane, Australia

**Keywords:** Adolescent, Obesity, Intervention, Self-determination theory, Physical activity, Dietary intake, Attitudes

## Abstract

**Background:**

Current estimates place just under one quarter of adolescents in Australia as overweight or obese. Adolescence has been identified as a critical period for the development of obesity, yet despite this recognition, there is limited systematic research into or evaluation of interventions for overweight adolescents. Reviews have concluded that there is a substantive evidence gap for effective intervention, but physical activity, lifestyle change and family involvement have been identified as promising foci for treatment.

**Methods:**

This paper reports on the development of a staggered-entry, waitlist controlled clinical trial to assess the impact of a multidisciplinary intervention aiming to change the poor health trajectory of overweight adolescents and help them avoid morbid obesity in adulthood—Curtin University’s Activity, Food and Attitudes Program (CAFAP). 96 adolescents, aged 11–16 years, and parents, will attend twice weekly during an 8 week intensive multidisciplinary program with maintenance follow-up focussed on improving activity, food and attitude habits. Follow-up assessments will be conducted immediately after completing the intensive program, and at 3, 6 and 12 months post intensive program. Main outcomes will be objectively-measured physical activity, sedentary behaviour and activity behaviours; food intake (measured by 3 day diary) and food behaviours; body composition, fitness and physical function; mental and social well-being (quality of life, mood and attitudes), and family functioning.

**Discussion:**

This trial will provide important information to understand whether a community based multidisciplinary intervention can have short and medium term effects on activity and food habits, attitudes, and physical and mental health status of overweight adolescents.

**Trial registration:**

Australian New Zealand Clinical Trials Registry ACTRN12611001187932.

## Background

### Overweight during adolescence is a major international health priority

It is estimated that 20 to 25 per cent of adolescents in Australia are overweight or obese [1,2]. Changes to environmental and societal factors such as a decrease in physical activity, an increase in sedentary behaviour and the availability of high fat, high-energy food have been identified as contributing factors to the high rates of overweight and obesity [3]. Obesity is seen as problematic in adolescents as it is related to short-term and long-term medical and psychosocial problems. Short-term difficulties can include physical discomfort, hypertension, orthopaedic problems, sleep apnoea and increased risk of heart disease and Type II diabetes [3,4]. Psychosocial problems can include social isolation, decreased self-esteem and depression [5,6].

Adolescence has been identified as a critical period for the development of obesity [7]. Obese adolescents are more likely to become obese adults [8,9]. Longitudinal studies have indicated that individuals who remain overweight from adolescence to adulthood have lower economic, educational and social outcomes than normal weight peers even after controlling for initial socioeconomic factors [10].

Despite the recognition of adolescence as a critical period in physical and psychosocial development, there is limited good research into interventions for overweight adolescents. Glenny et al. [11] conducted an early meta-analysis of obesity treatment, and reviewed a total of 13 randomised controlled trials with obese or overweight children or adolescents. They only considered studies with a minimum follow-up of 12 months, as maintenance of change was viewed as an important factor in the success of this type of intervention. They concluded there was a significant evidence gap with physical activity, lifestyle change and family involvement identified as promising foci for treatment. More recently, Oude Luttikhuis et al. [12] conducted a Cochrane Review for treatment of obesity with both adolescents and children. They reviewed a total of 17 randomised controlled trials with a focus on lifestyle interventions targeting physical activity, diet or behaviour therapy oriented treatments specifically for adolescents. Overall they concluded that ‘…family-based lifestyle interventions with a behavioural program aimed at changing diet and physical activity and thinking patterns provide a significant and clinically meaningful decrease in overweight in … adolescents’ (p17). Whitlock et al.’s [13] review also concluded that the recent evidence supports the efficacy of comprehensive programs of moderate to high intensity (>26 h contact). A brief review of some of the supporting evidence is presented below (see also [14]).

### Importance of activity behaviours

An imbalance between energy intake and energy expenditure can lead to obesity [15]. A change in energy balance is required to achieve weight-loss outcomes, by either increasing energy expenditure through physical activity or reducing energy intake or ideally a combination of both. Achieving this outcome in adolescents is further complicated by the need to recognise that growth is still occurring. Kemper et al. [16] conducted a longitudinal study measuring body mass index (BMI) and lifestyle factors from the age of 13 until 27 years. Their results demonstrated a strong relationship between high levels of physical activity and low body mass. Berkey et al. [17] conducted a large one-year prospective study and found that increased physical activity was associated with decreases in BMI in girls and overweight boys. Furthermore, increases in sedentary behaviour were associated with increases in BMI in girls.

Overall, research supports the utilisation of physical activity as a key component in adolescent obesity treatment. Interestingly the results from Anderson et al. [18] suggest that targeting sedentary behaviour may significantly improve outcomes. Results from a study by Epstein et al. [19] support this proposition. In their randomised controlled trial with 8 to 12 year olds, participants were allocated to a treatment group which either targeted physical activity, sedentary behaviour or both. At 12 month follow-up the sedentary behaviour group had significantly greater weight reduction than the other two groups. These results have yet to be replicated with adolescent participants.

Both increasing physical activity and reducing time spent in sedentary behaviours are part of the best practice weight management strategies for overweight adolescents as recommended by the National Health and Medical Research Council of Australia [1]. Independent of weight loss, overweight individuals who have become more active show improved cardiovascular and metabolic health [20-22], improved psychosocial health [22,23] and are less likely to gain weight [24]. Moreover, regardless of weight status, being more active and being less sedentary is associated with more positive health outcomes [25,26].

### Importance of food behaviours

An intake of foods high in fat and high in energy has the potential to lead to passive over-consumption which can contribute to the energy imbalance that precedes weight gain [27,28]. A low intake of fruit and vegetables is also thought to be a key contributor to the development of overweight and obesity [15]. A reduction in energy intake, whilst maintaining sufficient nutrition to support normal growth and development, is well accepted as the most appropriate dietary approach for treating obesity in adolescents [1,12,29]. Available evidence supports interventions that provide education on nutritional and energy values of foods, and encourage a healthier and more balanced diet, whilst discouraging dieting or restrictive eating [12]. Interventions that target dietary modification alone appear to be less effective than those that combine dietary modification and physical activity [30].

There is limited literature comparing the nutritional intake of overweight or obese adolescents to general adolescent diets, with under-reporting by overweight adolescents a critical issue [31,32]. Whilst two studies have shown no difference in energy intake when the diets of healthy weight children are compared to overweight children, this was thought to be due to under-reporting [33,34]. Adolescents, typically, have poor intakes of fruits and vegetables, with over three quarters of girls not meeting the guidelines for vegetable intake and a similar proportion of boys not meeting the guidelines for fruit intake [35]. Conversely, over 99% of adolescents consume energy-dense, nutrient- poor foods every day (these foods are referred to as ‘extras’ in the Australian Guide to Healthy Eating [36] but are commonly known as ‘junk food’ and will be termed as such in this study for ease of relating to adolescents), contributing to over 40% of daily energy intake [37]. These behaviours have the potential to contribute to a positive energy balance, which promotes weight gain.

The literature does identify a number of eating behaviours that are clearly linked to weight status, although it is important to recognise that causal relationships have not been established. Behaviours like skipping breakfast [38], eating whilst watching television [39], eating away from parents [40], consuming junk food [27,41] and drinking sweetened or carbonated beverages [42] have all been linked to greater levels of overweight and obesity. There is consistent evidence to support involving the whole family in making lifestyle changes around these behaviours [1,12,29].

Independent of body weight, an improved diet in overweight adolescents, alongside a more active lifestyle, is likely to reduce long term risk of chronic disease [43]. In overweight individuals, positive changes in dietary behaviours have resulted in improved cardiovascular and metabolic outcomes, irrespective of weight loss [20].

### Importance of attitudes

Spear et al. [15] argued that whilst most effective treatments for childhood obesity included both dietary and physical activity interventions, behavioural aspects also need to be considered. Behaviour modification treatment involves utilisation of behaviour change strategies aimed at changing thinking patterns and actions, in relation to dietary intake, physical activity or other aims. Oude Luttikhuis et al. [12] identified 12 studies that explored behavioural modification treatment with adolescents and performed a meta-analysis with four studies that met the criteria for this type of analysis. The meta-analysis revealed significant reduction in BMI at the end of treatment and maintenance of weight changes at 6 month follow-up compared with controls. The strategies that appeared most beneficial in behavioural management techniques were monitoring, goal-setting and contingency management [15].

In addition, Daley et al. [44] conducted a randomised controlled study with obese children aged 11 to 16 years. Participants were assigned to: an exercise-plus-therapy condition, where motivation to change and behavioural modification were addressed whilst they engaged in aerobic exercise; an exercise condition, involving light body conditioning only; or no treatment condition. Participants in the behavioural treatment condition reported higher levels of self-esteem, although there were no changes to depression scores or BMI. Whilst behaviour therapy based treatment can produce positive outcomes, difficulties remain in relation to small weight reductions, maintenance and psychosocial problems. Brennan et al. [45] argued that cognitive behaviour therapy (CBT) may be important in improving interventions for obese adolescents. Brennan et al. [46] conducted a randomised controlled study where CBT for weight loss was delivered on an individual basis to adolescents with their parents. They reported positive changes in weight loss and psychosocial measures. The current evidence supports the proposal that short-term CBT, in addition to behavioural strategies to address physical activity and eating behaviour, can provide positive outcomes in relation to weight changes and psychosocial well being.

### Importance of family

Garn et al. [47] found that children of two obese parents had an 80% probability of being obese compared with a 10% chance for children of two lean parents. Whilst it is acknowledged that there is a genetic factor to the development of obesity, parental attitudes and behaviour also appear to have an impact [1]. Furthermore parents often remain responsible for the majority of food choices within the home for most adolescents [1].

Stewart et al. [14] reported that the expert consensus was that parents’ engagement in obesity treatment with children and adolescents was important, and this was endorsed in the best practice weight-management strategies for overweight adolescents as recommended by the NHMRC [1] and recent systematic review findings [48]. In support of this, Andrews et al. [49] assessed 201 mothers and found maternal attitudes, social norms and perceived behavioural control predicted behavioural intentions and these in turn predicted parental monitoring of child food behaviours. Findings from the recent Loozit behavioural family lifestyle modification intervention, also support the importance of family involvement [50]. In their study, 150 overweight adolescents undertook an intense 7 week lifestyle modification intervention—with parents attending separate sessions focussing on practical support of behavioural change in adolescents and role modelling of healthy lifestyle behaviours. At 12 months post intervention, significant but modest reduction in BMI and improved psychosocial outcomes in the adolescents were reported [51].

### Theoretical framework

The underpinning theoretical framework that will be used to inform the intervention in the current trial is self-determination theory (SDT; [52]). SDT is a useful framework for identifying and explaining the motivational processes associated with behaviour change. Central to the theory is the concept of self-determined or autonomous motivation, characterized as performing behaviour out of a sense of volition, choice, and self-endorsed reasons as opposed to non-self-determined motivation which is characterised by acting for reasons of obligation, pressure, and extrinsic contingencies. A wealth of research indicates that individuals are more likely to persist with a behaviour if they endorse self-determined reasons for behaving. Self-determined motivation can be enhanced through environmental factors that promote satisfaction of basic psychological needs, particularly the need for autonomy [52,53]. Research has provided support for the efficacy of SDT in predicting physical activity and healthy eating behaviour [54-57].

In addition to SDT, theories of goal setting have been applied to explain motivation for a range of health behaviours including healthy eating and physical activity [58]. Despite reported successes, shortcomings still remain in the processes of goal setting. For example, research has yet to account for differences in goal content, goal motives, and psychological need satisfaction on behavioural persistence and goal attainment as outlined in SDT. Goal content refers to the ‘what’ of motivation, or an individual’s desires (e.g., to feel healthier), whereas goal motive refers to the ‘why’ of motivation, or an individual’s reasons for goal strivings (e.g., because my parent told me to exercise). Studies investigating goal content have demonstrated that considering goal content and goal motives strengthens the relationship between goal striving and goal attainment [59-61]. The current study seeks to integrate STD with goal setting theory by considering goal contents and goal motives as modifiable behaviours to enhance goal attainment.

### Pilot studies findings

The current intervention is based on our pilot studies. During 2009 the research team adapted a successful adolescent obesity tertiary hospital program (Princess Margaret Hospital ‘Fitmatters’ program) and delivered it within a university community context in school terms 2, 3 and 4. The adaption was based on the available evidence [12] and informed by the research group’s professional experience. In total 31 obese adolescents were invited to participate (mainly recruited by medical referral), 24 accepted and started the program and 16 completed the program [62]. Qualitative process evaluation found a high level of satisfaction with the program from both the adolescents and their parents and high attendance rates. No adverse events occurred. Qualitative impact evaluation identified critical family environment changes likely to impact on key behaviours, for example the removal of televisions from adolescents’ bedrooms. Quantitative evaluation found modest improvements in physical activity (increased moderate/vigorous and decreased sedentary) and food (increased fruit and vegetable and decreased junk food) for some, though not all, participants.

During 2011, qualitative studies were undertaken to examine barriers and facilitators to recruitment, retention in the program and maintenance of behaviours after the program. Focus groups were conducted with past participants (adolescents and parents separately) and potential participants. Interviews were conducted with key stakeholders including metropolitan and regional health professionals (medics, community nurses, physiotherapists, dietitians, psychologists), local community agencies (local council recreation and youth services officers), government agencies (Child and Adolescent Community Health Policy Unit, Department of Sport and Recreation, Public Health Units) and leading national researchers. The findings confirmed the general approach of Curtin University’s Activity, Food and Attitudes Program (CAFAP) and enabled refinement of recruitment processes, program content and delivery and maintenance phase contact. These contacts also supported provision of the program in local communities.

### Background summary

Given the large proportion of Australian adolescents currently overweight it is clear current practices are insufficient. In order to avoid the expected poor health trajectory, researchers [63-65] are recommending urgent evaluation of interventions targeting overweight adolescents.

Recent evidence suggests a lack of efficacy of a GP-delivered intervention for children [66]. On the other hand, a multi-component community-based overweight child program [67] has produced good results; and two multi-disciplinary community-based overweight adolescent programs [50,68] have also yielded promising results. Together these align with earlier expert panel recommendations for ‘comprehensive multi-disciplinary intervention… [to] encourage healthy behaviours while using techniques to motivate patients and families’ [15], p254. The most recent systematic reviews [12,13] and the recently released US Preventive Services Task Force Recommendation Statement [69] have confirmed this approach.

Oude Luttikhuis et al.[12] identified critical issues for future research including interventions for different levels of severity (CAFAP will target overweight and mildly obese), strategies for long-term maintenance (CAFAP will work with communities and families to identify sustainability factors), family characteristics which promote success (the process assessment will collect this), adolescent psychosocial factors which influence change (the broad evaluation framework will capture this), resource-effective methods for delivering interventions in different settings (CAFAP will evaluate this in metropolitan and regional community settings), and potential harm and benefits of intervention (CAFAP will monitor adverse effects as well as health outcomes).

Given the current evidence and strong recommendations for urgent action on this ‘global epidemic’ [12], this research will build on published evidence, recent pilot work and team experience to refine and implement a multidisciplinary family-centred community-based intervention intended to influence the physical activity, nutrition and psychosocial behaviours of overweight adolescents.

## Methods

### Design and aims

This study will use a staggered-entry, waitlist controlled clinical trial to assess the impact of a multidisciplinary intervention aiming to change the trajectory of overweight adolescents and thus help them avoid poor health in adulthood—Curtin University's Activity, Food and Attitudes Program (CAFAP). The specific aims of CAFAP are to:

· compare sedentary and moderate/vigorous activity before and after participation. It is hypothesised that sedentary behaviour will be reduced and moderate/vigorous activity will be improved after the program and these will be maintained at 3, 6 and 12 months post intervention;

· compare food intake and eating behaviours before and after participation. It is hypothesised that intake of fruit and vegetables will be increased and intake of junk foods will be reduced after the program and this will be maintained at 3, 6 and 12 months post intervention;

· compare physical status before and after participation. It is hypothesised that BMI, waist circumference, cardiovascular fitness, muscle endurance, strength and power will be improved after the program and this will be maintained at 3, 6 and 12 months post intervention;

· compare mental well-being before and after participation. It is hypothesised that mental well-being will be improved after the program and this will be maintained at 3, 6 and 12 months post intervention; and

· compare perceived quality of life before and after participation. It is hypothesised that quality of life will be improved after the program and this will be maintained at 3, 6 and 12 months post intervention.

The study will also explore the influence of adolescent age, gender, baseline status, autonomy support, autonomous motivation, goal attainment and conflict, parental factors and family function on changes in adolescent outcomes. Process evaluation will also be conducted to assess program fidelity, satisfaction and adverse outcomes.

### Participants and recruitment

Adolescents (n = 96, 11–16 years of age) will be recruited via the health system, the education system and from the general community. Paediatric specialists, allied health professionals at a tertiary children’s hospital, general medical practices close to the study and nurses in schools close to the study will be informed about the study and asked to identify potentially suitable adolescents. Community newspapers and radio mass media will be used to inform the general community of the study. Volunteers, who meet the inclusion and exclusion criteria and are aware of the full study responsibilities, will be screened by a medical practitioner to ensure they are medically suitable to participate. Inclusion criteria are: males and females aged 11–16 years, BMI higher than the 85^th^ centile on the standard Centers for Disease Control (CDC) BMI-for-age growth charts [70] (includes children who are typically classified as overweight or obese), and have passed medical screening. Children will be excluded if: their obesity is due to identified genetic, metabolic or endocrine disease, they are undergoing treatment for psychiatric disorders, they reside remotely or are unable to attend twice weekly sessions at the designated community intervention locations, or they are assessed unsafe to participate by GP or Paediatrician.

### Sample size

A sample size of 96 is required at post test in the intervention group to detect a 20% difference in the outcome variables at 80% power and 5% level of significance. A small effect size (Cohen’s d = 0.2) is assumed for studies on behavioural effects due to the influence of extraneous variables and the subtleties of human performance.

### Intervention

The intervention consists of an intensive 8 week multidisciplinary program focussed on improving activity, food and attitude habits with tapered follow-up for 12 months. The intensive phase will run within school terms and be delivered by community health professionals following training in the philosophy and approach of the program. Groups of 12 to 15 adolescents and their parents will attend twice weekly. On each occasion adolescents will participate in a 45 minute exercise class involving aerobic, strength and skill stations. Adolescents will also participate in hour long education sessions covering healthy activity, healthy eating, energy balance, food labelling, preparing meals and snacks, goal setting, problem solving, dealing with mood, family activity and eating and relationship with family. Parents will participate in education sessions covering the same issues. They will also have sessions on understanding adolescence, providing support, relationships with adolescents, community resources, food budgeting and a supermarket visit. There will also be informal support from other parents and staff in ‘walk and talk’ sessions. The intensive phase will conclude with a healthy cooking celebration. During the 12 month follow-up maintenance phase, participants will be contacted, with decreasing frequency, to prompt individual activity and eating goals.

### Study design

**Figure 1:**
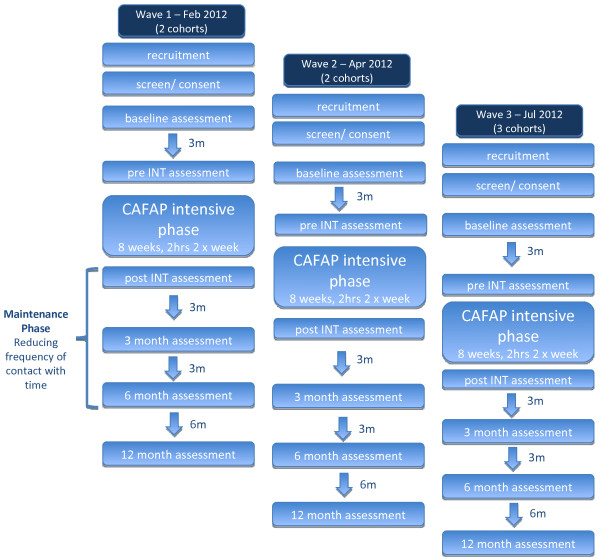
**Trial flowchart.** This figure provides an overview of the participant flow for the cohorts in each wave of the study.

CAFAP will be evaluated using a multiple cohort, staggered-entry, waitlist controlled clinical trial design (see Figure 1). Participants will be recruited and given an initial assessment then reassessed after 3 months waiting (just before starting the program). The dual pre-participation assessments will provide a within-subjects control period. Follow-up assessments will be conducted immediately after completing the intensive program (3 months after second pre-program assessment) and at 3, 6 and 12 months post program.

Cohorts of 12–15 overweight adolescents and their parents will be progressively recruited over the study period. Cohorts are expected to start in February 2012, April 2012 and July 2012, and continue until early 2014. The staggered start will control for external seasonal and public event confounders to intervention effects.

### Primary outcome measures for adolescent

#### Physical activity/inactivity

Time spent in sedentary, light, and moderate-to-vigorous intensity physical activity will be assessed over 7 days using an Actical accelerometer worn on the hip. The MiniMitter Actical is a widely used and validated accelerometer in studies of children and adolescents [71-73]. Seven days of accelerometer measurement are recommended for the purposes of acceptable measurement of moderate to vigorous PA [72]. Intensity will be determined using the Actical cut-offs reported by Colley et al. [73]. Total weekly time in all activity intensities will be calculated as well as weekend and after school weekday intensity times.

#### Food intake

Food intake will be assessed using a 3-day dietary record using weighed or household measures. Collecting dietary data from overweight and obese adolescents and minimising underreporting poses many challenges. There is insufficient evidence in the literature to support the use of one dietary measurement tool over another [31,32,74]. Given the small group size, variation in daily adolescent diet and burden of dietary data collection, a three day food diary has been selected as the most appropriate tool as it will provide data on meals and snacks which will inform the intervention [31]. Adolescents will be asked to record everything they eat and drink over a period of three days, including one weekend day. They will be provided with written directions and verbal instruction from the research dietitian regarding estimating portion size. On returning the dietary record, the dietitian will clarify details and amounts of foods. The number of serves of fruit and vegetables will be determined according to the Australian Guide to Healthy Eating serve sizes [74], and junk food will be identified according to the Food Criteria System devised by Rangan et al. [37] and classified into 600 kJ servings as per the Australian Guide to Healthy Eating [36]. The diet records will be analysed for specific nutrient intake using the AUSNUT database and Foodworks Professional edition version 3.02 software. Data will be extracted for intakes of total energy, macronutrients and percentage contribution to energy intake, as well as micronutrients. All nutrition analyses will be completed by a dietitian.

### Secondary outcome measures for adolescent

#### Sedentary and physical activity behaviours

Weekly frequency and school day/weekend day duration of participation in common sedentary behaviours (TV viewing, playing electronic games, using a computer) and moderate/vigorous physical activity (sports, dance, active play) will be assessed using questions drawn from the Western Australian Child and Adolescent Physical Activity and Nutrition Survey and the Healthy Kids Queensland Survey [75,76].

#### Food behaviours

Specific food behaviours that have been shown in the research to be associated with unhealthy outcomes and greater levels of overweight status, such as eating in front of the television or missing breakfast, will be assessed using questions drawn from the NSW Schools Physical Activity and Nutrition Survey [77] and the Healthy Kids Queensland Survey [75,76].

#### Physical status-BMI and waist circumference

Weight, height and waist circumference measurements will be made using calibrated scales, stadiometer and inelastic tape measure and standardized protocols. Body mass index will be calculated and age and sex adjusted BMI z-scores (calculated against CDC reference data) will be determined to enable comparison with state-wide surveys relevant to the Australian context (Western Australia and Queensland).

#### Physical status-fitness

Cardiovascular fitness will be assessed using the modified incremental shuttle walk test [78]. This test is based on the widely used incremental shuttle run test designed for healthy individuals [79] with the reduction of shuttle distance from 20 m to 10 m to provide a lower demand start to the test for those with health problems. Participants walk/run between markers in time with beeps. The beep interval decreases every minute, requiring increases in walking velocity. The test is terminated when the participant can no longer reach the end of the 10 m in time with the beep. In obese adolescents test-rest reliability is very high (ICC = 0.92) and validity has been demonstrated with strong correlations with other aerobic indices including maximal oxygen uptake in bicycle ergometer tests (r = 0.79) [80].

#### Physical status-muscle performance

Strength of the quadriceps, biceps and deltoid muscles will be assessed using standard ‘break’ manual muscle testing protocols [81]. ‘Break’ tests require the participant to hold a position while the tester applies progressive resistance until the position is broken. Peak force will be recorded using a force transducer. All muscle testing will be performed on the non-dominant limb and each test will be performed 3 times, with an average score used for analysis. Hand-held force transducer tests of muscle strength have been shown to have good criterion validity in comparison to laboratory dynamometers, with good intra-tester and inter-tester reliability [82].

Lower limb muscle power will be assessed using a vertical jump protocol [83]. Three measures are taken and the highest recorded [84,85]. Vertical jump has been shown to be appropriate for measuring the explosive power of the lower limbs in both sedentary and athletic populations [85] and has been used to determine power outputs in obese adolescents [86].

Abdominal muscle endurance will be tested via a partial curl-up test of the Canadian Standardised Test of Fitness [87]. Measurement will be taken of the number of curl-ups performed correctly in 1 minute. This is a reliable and valid test for measuring the dynamic endurance of abdominal muscles [88].

#### Mental health

The primary mental health outcome measure will be depressive symptoms assessed using the Moods and Feelings questionnaire (MFQ; [89]). The MFQ is a 33 item self-report measure which looks at depressive symptoms in children and adolescents. It has good test-retest reliability [90] and has been validated with a clinical youth population [91].

#### Perceived autonomy support and autonomous motivation

The Perceived Autonomy Support Scale for Exercise Settings (PASSES; [92]) and the Perceived Environmental Supportiveness Scale (PESS; [93]) will be modified to measure perceived autonomy support, structure, and involvement from parents and instructors in regard to physical activity and healthy eating behaviours. Autonomous motives for physical activity behaviours will be measured using the revised Behavioural Regulations and Exercise Scale (BREQ-2; [93]) and the integrated regulation scale for exercise behaviour [94]. Autonomous motives for healthy eating behaviours will be measured using an adapted version of Mullan, et al.’s BREQ [95] and Ryan and Connell’s [96] perceived locus of causality for diet (PLOC; [97,98]) and the integrated regulation scale for exercise behaviour [94].

#### Goal attainment and goal conflict

Adolescents will be asked to report their overall goals to achieve between each data collection point (end of 8 week program and 3, 6, and 12 month follow-up) in the areas of physical activity, sedentary behaviour, and healthy eating. The amount and frequency of a behaviour (e.g., be active for 30 minutes (moderate to vigorous) four times a week) will be detailed in the overall goal to provide an outline for breaking long-term goals into smaller more manageable weekly goals. Adolescents will be provided weekly goal setting sheets for use during the 8 week program and one year post-program period. Weekly goal sheets will include the following information for each goal area: specific weekly goal (e.g., to be moderately active for 15 minutes 4 times/week and do more than 8000 steps 4 times/week); challenge rating; goal content (i.e., intrinsic—health; extrinsic—appearance); goal motive (autonomous—inherent enjoyment; controlled—external pressure); goal details for each day of the week (activity, frequency, time of day to perform activity); daily goal steps and actual steps; weekly and overall goal progress. Participants will respond to the challenge rating using a scale ranging from 0 (*easy*) to 10 (*impossible*) to indicate their perceived goal difficulty. Goal progress ratings will be completed one week after goals have been set using a 0 to 10 scale. Weekly goal progress scores for each goal area will be summed across each week to form a single index for weekly goal attainment [99]. Overall progress scores will be summed across all goal areas to form a single index for overall goal attainment [99]. During adolescents’ first goal setting session, they will be asked to list their four most important life goals and rate how much they believe these goals will enhance or interfere with the goals they set for physical activity, sedentary behaviour, and healthy eating. At each follow up data collection, adolescents will be presented with their four life goals stated at baseline and asked to indicate how much these goals interfered with their behaviour change goals for physical activity, sedentary behaviour, and healthy eating [100,101]. Responses will be indicated using a scale from 0 (made goal achievement very difficult) to 10 (made goal achievement very easy). Scores will be summed across all four life goals to form a single index for goal conflict.

#### Quality of life

Quality of life will be assessed with the Paediatric Quality of Life—Teen Report (PedsQL) [102]. The PedsQL is a 23-item self-report quality of life measure for 13–18 year olds. This measure has been demonstrated to have good validity and reliability [102,103].

### Secondary outcome measures for parents

#### Parental sedentary and physical activity behaviours

As per the adolescents, parents will complete a brief questionnaire on sedentary and physical activity behaviours using questions drawn from the Western Australian Child and Adolescent Physical Activity and Nutrition Survey and the Healthy Kids Queensland Survey [75,76].

#### Parental mental health

Parents will complete the adult version of questionnaires covering the moods and feelings area (DASS-21). The DASS-21 is a short-form of the Depression, Anxiety and Stress Scale (DASS; [104]). The DASS has been shown to reliably distinguish between symptoms of depression, anxiety and stress in clinical [105] and non-clinical samples [104]. The DASS-21 has been demonstrated to have acceptable reliability and validity [106].

#### Parental perceived autonomy and autonomous motivation

The adapted version of the PESS [93] and PASSES [92] used for adolescents, will be modified to measure parents’ perceptions of autonomy support, structure, and involvement provided by the instructor. The scale will be adapted to assess parents’ perceptions in regard to supporting their adolescents’ behaviour changes for physical activity and healthy eating. Autonomous motives for supporting adolescents’ physical activity will be measured using the revised BREQ-2 [93] and the integrated regulation scale for exercise behaviour [94] previously described for adolescents. Autonomous motives for supporting adolescents’ healthy eating behaviours will be measured using an adapted version of the PLOC [97,98] and integrated regulation scale for exercise behaviour [94] used to measure adolescents’ autonomous motives.

#### Parental goal setting and goal conflict

The overall and weekly goal setting sheets previously described for adolescents will be adapted for parents to use in setting their own goals to help support adolescents’ behaviour change goals. Goal progress and goal challenge scores will be reported using the same format previously described for adolescents [99]. Goal conflict sheets for parents will be adapted from those previously described for adolescents in order to assess parents’ goal conflict regarding the goals they set for supporting their adolescents’ physical activity, sedentary behaviour, and healthy eating [100,101].

#### Family functioning

Parents will also complete the general functioning scale of the McMaster Family Assessment Device (FAD; [107]). The FAD is a 53 item self-report which assesses family functioning on five subscales; problem-solving, affective responsiveness, affective involvement, behaviour control and general functioning. The FAD is suitable to be administered to all members of a family over 12 years, and has adequate internal consistency, test-retest reliability and concurrent validity [107,108].

### Program evaluation

There is substantial agreement that interventions for overweight young people need to be evaluated in terms of process and outcome and not just immediate impact [109] to understand not only how much the intervention works but why and for whom.

#### Process evaluation

A broad process evaluation will be conducted in line with the recent framework for mandatory evaluation to ensure well informed public policy decisions [110] and recommendations by the recent Cochrane review for obesity interventions [12]. Questionnaire surveys of participants, focus groups of participants and program facilitators are anticipated [111]. Program fidelity will be assessed through observations of sessions, review of program notes and focus groups with program staff [112]. Program dose will be assessed by attendance records. Barriers to participation will be explored through adolescent/parent surveys including reasons for non-attendance and gaining reasons for drop out from those who do not complete the program [113]. Participant satisfaction will be assessed with a validated tool [114] along with focus groups. Monitoring will also be conducted for potential adverse effects, specifically: changes in linear height and psychological well-being, as recommended [12].

#### Impact evaluation

Immediate impact of the intervention will be assessed by comparing the changes over the 3 months between pre-program assessment and immediately post-program assessment with any changes over the 3 months waitlist period between initial assessment and pre-program assessment. The primary impact measures will be changes in: 1) leisure time spent in light and moderate/vigorous physical activity; 2) leisure time spent in sedentary activity; 3) number of weekly serves of fruit and vegetables and 4) number of weekly serves of junk foods. Secondary impact measures will include: physical status (BMI z-score, waist circumference, cardiovascular fitness, muscle performance), mental health, perceived autonomy, sedentary and diet behaviours, parental mental health and family functioning.

#### Outcome evaluation

The longer term changes in behaviour (same variables as short term impact), as well as physical and mental well being and family functioning, will be assessed by comparing changes between assessments at baseline and at 3, 6 and 12 months post intervention.

#### Expected program outcomes

The primary program outcomes are expected to be improved adolescent behaviours including: an increase in physical activity (~10% change); reduction in sedentary behaviours (~10% change); an increase in fruit and vegetable serves (~10% change); a reduction in junk food serves (~10% change). It is anticipated there will be improved physical status; improved mental health including reduced symptoms of depression; improved quality of life and improved family functioning. Improved parental mental health is also expected.

### Trial flow

Following medical screening, participants will be provided with a description of the study and given an opportunity to have responsibilities, risks and benefits of participation clarified. After informed consent/assent from parent and adolescent, baseline study entry assessments are completed. Participants then enter a 3 month waitlist period and are then reassessed pre-program. Participants will complete the 8 week intensive phase of the intervention and be re-assessed. During the maintenance phase participants will be assessed at 3, 6 and 12 months post intensive phase. Each cohort of 12 to 15 adolescents will follow this pattern, with new waves of cohorts starting with each new school term. Two cohorts will start in Waves 1 and 2, with three cohorts starting in Wave 3 (See Figure 1).

### Analysis

Process evaluation will use descriptive statistics and qualitative analysis. Impact and outcome evaluation will use a multi-level mixed modelling approach for the hierarchical data collected over the 18 month (waitlist, intensive program and maintenance) period (repeated measurements of individuals nested within families). The degree of change in outcomes over time periods will be examined for associations with adolescent age, gender, baseline status, autonomy support, autonomous motivation, goal attainment and conflict, parental factors and family function, by examining time interactions with these variables. All change estimates will be presented as adjusted mean change with corresponding 95% confidence intervals. Non-normally distributed data will be appropriately transformed as necessary, and statistical models checked for unduly influential outliers. All data analysis will be performed utilising current statistical software (Stata/IC 12.0 for Windows, Statacorp). A critical alpha level of 0.01 will be used to balance type 1 and type 2 errors.

### Ethics

Ethical approval for this intervention has been received from Curtin University Human Research Ethics Committee (HR105/2011).

## Discussion

Adolescent obesity is recognised as a major health problem internationally due to the anticipated health burden as these individuals age. It is widely believed that obesity is a multifaceted construct requiring a multidisciplinary intervention. The available efficacy evidence suggests that activity, food and attitude interventions are effective independently but that combined interventions are likely to be more effective. Trials of a pilot multidisciplinary intervention have achieved encouraging results and qualitative pilot studies have suggested ways to improve the immediate and sustained impact of the intervention. This study will evaluate CAFAP, a revised community based program incorporating best practice components in an integrated intervention for overweight adolescents and their families.

### Implications

This trial will provide critical information to understand whether a community based multidisciplinary intervention can have short and medium term effects on activity, food and attitude habits and physical and mental health status of overweight adolescents. The primary outcomes are adolescent moderate/vigorous physical activity, light activity, sedentary time as well as fruit and vegetable intake and junk food intake, which are important for adolescent health regardless of weight status. Secondary outcomes include adolescent physical status, mental health and parental mental health and family functioning. CAFAP aims to shift habits and thus have a substantial impact on medium to long term health.

### Trial registration

This trial is registered in the Australia and New Zealand Clinical Trials Registry # ACTRN12611001187932.

## Competing interests

The authors declare that they have no competing interests.

## Authors’ contributions

All authors have contributed substantially to this protocol. LMS conceived the study, contributed to the study design, the physical activity domain and drafted the manuscript. KLS and DK contributed to the nutrition domain; AAF, MD, AMF and MSH contributed to the psychosocial domain; TO and RAA contributed to the physical activity domain; AM contributed to the program design; and AJS contributed the analysis plan. All authors have read and approved the final manuscript.

## Pre-publication history

The pre-publication history for this paper can be accessed here:

http://www.biomedcentral.com/1471-2458/12/471/prepub
